# Family Economic Burden of Elderly Chronic Diseases: Evidence from China

**DOI:** 10.3390/healthcare7030099

**Published:** 2019-08-21

**Authors:** Xiaocang Xu, Xiuquan Huang, Xiaolu Zhang, Linhong Chen

**Affiliations:** 1 Upper Yangtze River Economic Research Center/School of Economics, Chongqing Technology and Business University, Chongqing 400067, China; 2 Upper Yangtze River Economic Research Center, Chongqing Technology and Business University, Chongqing 400067, China; 3 School of Economics, Chongqing Technology and Business University, Chongqing 400067, China; 4 School of Mathematics and Statistics, Chongqing Technology and Business University, Chongqing 400067, China; 5 School of Public Administration, Sichuan University, Chengdu 610065, China

**Keywords:** Elderly chronic disease (ECD), Economic burden, ECD-family, Health service utilization, Workforce participation

## Abstract

Chronic diseases among the elderly and their huge economic burden on family have caught much attention from economists and sociologists over the past decade in China. This study measured the economic burden of elderly chronic disease (ECD) in families using the China Health and Retirement Longitudinal Study (CHARLS) data set from Peking University (China). We studied some aspects of this burden, including health-service utilization, out-of-pocket expenditure on inpatient and outpatient, total family expenditures on items, and labor force participation rates of family members, etc. Some interesting things were found, for example, the additional annual expenditure on inpatient care (per member) in ECD-families was 37 to 45 percent of the annual expenditure in the control group; the labor-force participation rate in ECD-families was 2.4 to 3.3 percent of points lower than in the control group.

## 1. Introduction

The latest data from global cancer research predicted that the number of new chronic disease among the elderly (ECD) cases all over the world will boost from 12.7 million in 2008 to 21.4 million in 2030 [1]. Since ECD maybe cause disability and shorten life expectancy, it thereby increases the economic and social burden of treatment and prevention on families. Therefore, research on ECD and assessment of the ECD burden among the families of the elderly can provide empirical evidence for all the countries to achieve the control and prevention of ECD [2,3,4].

In China, improvements in health service, accompanied by growth in economic development, have caught much attention from economists and sociologists over the past decade. In China, life expectancy at birth rose nearly 51% from 49 in 1950 to 74 in 2016. However, outside of these health and economic gains, China’s health service policy makers face the challenge of chronic diseases and a significant disease burden from elderly chronic disease (ECD), which has high death and disability rates and requires expensive hospitalization costs. ECD incidence has been increasing rapidly, due to the combination of risk factors such as diet, tobacco, etc. in the elderly [5,6,7]. 

As a result, ECD incidence and ECD-related mortality were higher than those in developed countries such as the UK and USA [8,9,10]. Furthermore, China is becoming an ageing society, where the elderly aged 60+ make up more than 12 percent of the total population, above the 10 percent threshold for an aging society set by the United Nations [11], followed by the significantly increased incidence and disability rate of ECD among the elderly. As a consequence, ECD imposes huge economic burden on the family [12].

Several previous studies on the family economic ECD burden have appeared but do not sufficiently describe the issue, especially in China [13,14,15]. Firstly, their measures of the economic ECD burden were confused with one single disease and its comorbidities. Unadjusted measures of the economic burden of any single disease may be skewed upwards, since common risk factors that increase the risk of comorbidities occur. For instance, obesity or smoking may lead to diabetes or heart disease [16]. Secondly, the differences in education status and population structure among families may affect health-service expenditure and other behavior arrangements [17]. Finally, there is the impact of chronic diseases of the elderly (ECD) on households not affected by chronic diseases of the elderly (ECD), reducing employment and income through slower overall economic growth [18]. In view of this, this paper is particularly concerned about families including a member with elderly chronic disease (i.e., ECD-family), and comparing them with a similar family without a member with elderly chronic disease (ECD). 

This study measured the economic burden of elderly chronic disease (ECD) in Chinese families using the China Health and Retirement Longitudinal Study (CHARLS) data set from Peking University (China). The ECD burden is undoubtedly severe in China since there is a low level of public finance funding for health services (increased from 0.9% to 1.2% of Gross domestic product (GDP) over the past two decades) and underdeveloped insurance markets have forced ECD-families to use out-of-pocket payments [19]. We studied some aspects of this burden, including health-service utilization, out-of-pocket expenditure on inpatient and outpatient health-service, total family expenditures on items other than health-service, reliance on borrowing or asset sales, and labor force participation rates of family members. This paper also discussed the impact of other family members when the ECD patients are living at home, including their labor force participation, health-service use, and medical expenses [20]. 

## 2. Materials and Methods

### 2.1. Concept Definition and Variable Selection

#### 2.1.1. Definition of ECD-Family

An ECD-family is defined as having at least one member with elderly chronic disease (ECD): (a) currently living with elderly chronic disease (ECD); or (b) being in hospital due to elderly chronic disease (ECD) in the year preceding the survey, whether or not currently alive. In the CHARLS data, approximately 54% of elderly chronic disease (ECD) cases were reported to be hospitalized in the year preceding the survey, which is lower than the annual hospitalization rates of elderly chronic disease (ECD) patients reported in developed countries such as Australia (about 70%) for which similar data are available [21]. Families in the control group did not fit this definition.

#### 2.1.2. Matching Variables

ECD-family and control families were matched on several indexes, including the educational status of the family, house-type, the ownership of land, sanitation type, the main source of sustenance, population structure (number of children, young and the elderly, proportion of family members that are women), caste (whether scheduled caste or tribe or not, or other backward classes or not), rural/urban status and whether family members have social insurance or private insurance.

#### 2.1.3. Outcome Variables

(a) Health service utilization of families. It consists of two categories: healthcare utilization and health expenditure. Healthcare utilization variables included hospitalization rate per family member in the one year preceding the survey, the length of hospital stay per family member, and outpatient visits per family member in the 15 days preceding the survey. Health expenditure variables included family out-of-pocket medical expenses per member for outpatient care in the 15 days preceding the survey, and family expenses per member for hospital (inpatient) care in the one year preceding the survey.

(b) Non-Medical consumption expenditure of families. Costs of treatment and loss of income related ECD may affect the expenditure on items other than healthcare in the ECD-family [22]. Family consumption expenditures, and net of healthcare expenditures per member were used to capture this effect for the first 15 days of the survey.

(c) Out-of-pocket healthcare expenditure. To assess the degree of ECD-related financial stress faced by families, two variables were used to illustrate this: whether families borrowed funds or sold assets to finance inpatient care (one year before the survey) and whether families borrowed or sold assets to finance outpatient care in the 15 days preceding the survey [23,24].

(d) Workforce participation among adults. We compared adult workforce participation rates (the number of members aged 45 years and over who are currently working, divided by the total members in the family aged 45 years and over) among ECD-family members and members of matched control families.

(e) Impact on members without ECD in ECD-families. In an ECD-family, the ECD burden can be felt through changes in workforce participation, healthcare expenditures and non-medical consumption for members without ECD [25,26,27,28]. Therefore, we compared family-level outcomes (number of outpatient visits, hospitalization rate, and health expenditures) of members without ECD in the ECD-family and control families. 

### 2.2. Methods and Steps 

Propensity score matching (PSM), which was used first by Rosenbaum and Rubin [29], was used to compare the results for the ECD-family to the matched-control family or unmatched-control family. This usually consists of three steps. 

In the first step, the propensity score (the likelihood that the ECD-family) is predicted based on the education and demographic characteristics of the family. The following Logit model (1), with reference to Ajay et al. [30], was estimated:

(1) $$P\left(\frac{C_{i}=1}{X_{i}}\right)=\frac{e^{\beta X_{i}}}{1+e^{\beta X_{i}}}$$

Here $C_{i}$ means whether family i includes a member with Elderly chronic disease (ECD). The vector $X_{i}$ represents the family population and education characteristics, and β is the vector of the parameters to be estimated.

In the second step, ECD-families were matched with control families with similar propensity scores using R. To check for balance, for each covariate used in the regression model that produced the propensity scores, we used the t-test to compare the mean between ECD-families and those in the control group.

In the end, robustness checks were implemented to evaluate the results of many matching methods, such as the nearest neighbor method and the hierarchical method. By estimating the results after excluding data, 1% hospitalized ECD-patients with the highest out-of-pocket costs were treated to reduce the risk of a few outliers influencing the outcome; by excluding any family in which at least one person has died because of varying degrees of underreporting of deaths in family surveys. Furthermore, to address the risk of upward bias in the estimates of hospitalized ECD cases used in our definition of ECD-families, we estimated another set of outcomes by including hospitalization indexes in the propensity score equation when generating the propensity score.

### 2.3. Data Sources

The main database used in this study was from the China Health and Retirement Longitudinal Study (CHARLS), which has received funds from the World Bank, Peking University (China), and the Behavioral and Social Research Division of the National Institute on Aging (China). CHARLS, covering the evaluation of economic, social, and health status of citizen, is a nationally representative longitudinal survey for people aged 45+ in China. CHARLS used multi-stage stratified PPS sampling and a software package” CHARLS-GIS” (Peking University, China) to make sampling frames. Its questionnaire covered the following information: population structure, family structure, health status, health service and insurance, work status, retirement and pension, income and consumption, and assets (individual and family), etc. The CHARLS interviewees using face-to-face form were followed up every two years. The latest survey (2015) included about 10,000 families and 17,500 individuals in 150 districts of 28 provinces [31]. 

It is the most reliable source of national-level information on family health service utilization although our identification of ECD-families relies on self-reporting, which can lead to inaccurate estimates of ECD cases. For example, in the CHARLS data we used, the prevalence of ECD cases was 51.2%, which is close to the 53.8% prevalence estimate reported by China health statistics yearbook (CHSY) for people aged 60+. In addition, the estimated annual hospitalization rate of ECD-families in CHARLS is 54%, which is lower than in developed countries such as Australia (70%). However, although the absolute value of the coefficient estimate is low, the direction of the result is similar.

## 3. Results

### 3.1. Summary of Variables

Table 1 showed that the health care use patterns and out-of-pocket expenditure measures of matched and control families affected by elderly chronic disease (ECD) are more closely related to each other than are mismatched controls. 

Therefore, simply comparing ECD-families with randomly selected families that do not report ECD may result in an upward bias estimate of the economic burden of elderly chronic disease (ECD). 

In addition, a wide range of family socio-economic and demographic characteristics was used for matching from the five aspects of age structure, level of schooling of household head, sanitation, caste and geographic region. The sample means of matched-control family were quite close to the ECD-family going through the relevant t statistics (omits here).

### 3.2. Effects of ECD on Family Health Service Utilization

#### 3.2.1. Results of Effects of ECD on Family Health Service Utilization

Effects of ECD on family health service utilization were shown in columns 2 to 5 of Table 2. Direct comparison of ECD-family and control family using the nearest neighbor and stratification method (columns 2 and 3), excludes all families experiencing at least one death before, and excludes the highest 1% of ECD-related inpatient family expenditures. 

ECD-family received 25.7 to 27.6 additional hospitalization rate per 100 members per year and 6.8 to 9.9 additional outpatient visits per 100 members per 15 days prior to the survey, compared with the control group. The average family member affected by ECD also spends more time in the hospital. In the 15 days before the survey, family members without ECD (ECD-family) had fewer outpatient visits per capita (two to three visits per 100 family members) than family members in the control group. Per capita, outpatient visits for non-primary health conditions in the 15 days before the survey were also lower in ECD-family than in the control group, with 3.8 to 5.6 visits per 100 members.

#### 3.2.2. Robustness Check

We also conducted a separate set of analyses that included indexes of hospitalization as matching variables to generate propensity scores (in addition to core socio-economic and demographic characteristics). Thus, the large number of families whose members were hospitalized for reasons other than ECD was used as a control and reduced estimates of the economic impact of ECD. As shown in Table 3.

### 3.3. Effects of ECD on Family Consumption and Workforce Participation

#### 3.3.1. Results of Effects of ECD on Family Consumption and Workforce Participation

The effects of ECD on family consumption and workforce participation were presented in columns 2 to 5 of Table 4. Out-of-pocket medical expenses paid were significantly higher in families compared with ECD control, between CNY 4577 and CNY 5439 per inpatient expenditure per member in the year before the survey and CNY 67 and CNY 86 per member in the 15 days before the survey per capita outpatient visits. Compared to the control group, the health care expenditures of individuals other than ECD patients and non-primary health conditions in ECD-family were lower. ECD-family spent less per family member on non-medical expenses than the control group, with each family member spending between CNY 28 and CNY 86 on the first 15 days of the survey, although statistical differences were not always zero.

Compared with the control group, ECD-families were much more dependent on out-of-pocket spending for treatment: between 33.6 and 39.3 percent of inpatients in the year before the survey; the expenditure on outpatient services during the 15 days before the survey was 3.2% to 4.2%. 

Compared with the control group, the labor-force participation rate of family members aged 16 years and older was 2.41 to 3.22 percent lower in ECD-families. When ECD patients were excluded from consideration, adult members of ECD-families had a higher labor force participation rate of 0.82% to 1.92%, but the results were not statistically significant. 

#### 3.3.2. Robustness Check

However, as shown in Table 5, although our estimates of family financial burdens are low, they do not affect the basic direction of our conclusions.

## 4. Discussion

Improvements in health, accompanied by the growth in the economic development, have caught much attention from the economists and the sociologist over the past decade in China. This paper has studied the family economic burden of elderly chronic diseases in China based on CHARLS data. Some interesting things were found.

First, the effects of ECD on family health service utilization. ECD-families experienced a greater rate of hospitalization and outpatient visits than the control group. Given China’s low health insurance coverage, it is not surprising that ECD-families find a huge burden of out-of-pocket spending. The additional annual expenditure on inpatient care (per member) in ECD-families was 37 to 45 percent of the annual expenditure in the control group. About 35 to 43 percent of the expenditure of the average ECD-family is spent on out-of-pocket inpatient and outpatient care. Compared with families with lower education status (54%), families with higher education status spend a higher proportion of total expenditures on out-of-pocket medical care (61%).

Second, effects of ECD on family consumption. Out-of-pocket costs associated with ECD treatment and any loss of income affect non-medical spending by ECD-families. We expect that when some family members have ECD, the non-medical consumption of family will be lower unless the family can effectively guard against the related financial risks. We found that ECD-families spend less per capita. The estimated negative impact of net health expenditure on per capita consumer expenditure suggests that Chinese families are not adequately protected from the financial risks of ECD compared to controls. However, this reduction is much less than the estimated additional out-of-pocket costs of inpatients (CNY 4577 and CNY 5439). The same conclusion applies to families of different education status. Compared with the control group, families with high education status had lower non-medical expenses and higher inpatient out-of-pocket care costs. Similarly, the non-medical expenses of families with low education status lower than those of the control group, but the annual inpatient costs were higher. As a result, families rely on other ways to protect their non-health expenses from ECD-related expenses.

Families also cope with the ECD cost by increasing the burden of other members on unaffected members [32]. Once ECD patients were excluded from the analysis, the number of outpatient visits and out-of-pocket medical expenses of affected family members was significantly lower than those of the control group. However, when the data are broken down by education status, the results apply only to families with higher education status. We also compared the use and cost of health care for non-major diseases (i.e., diseases other than stroke, heart disease, injury, and diabetes), assuming that the occurrence of serious diseases would reduce the use of health care for the former. We found that the rate of outpatient visits per capita for non-primary diseases in ECD-families decreased by three to five times per 100 people compared to the control group, even when the data were divided into two groups.

Finally, the effects of ECD on workforce participation. With more than 90% of China’s workers mostly in the informal sector and limited social security benefits, the loss of income from absenteeism by patients and their caregivers is likely to rise. Overall, the labor-force participation rate in ECD-families was 2.4 to 3.3 PCT points lower than in the control group. However, in ECD-families, the labor force participation rate was 0.7 to 1.9 PCT points higher in those without ECD than in the control group, although the results were statistically close to zero. These estimates do not show an impact on working hours or income but suggest that the negative impact of ECD on family income may be partially offset by an increase in Labor force participation among healthy family members. Even here, the decline in adult labor force participation was much larger in families with low education status (as compared to the control group) than in families with high education status. 

Our research has several advantages. Matching families affected by ECD with “control” families (those unaffected by ECD) on huge observable education and demographic characteristics can reduce the confusion caused by non-random allocation of ECD. Our results suggest that the economic ECD burden-whether it is public subsidies or out-of-pocket spending by families-may not be as great as people conclude in the absence of a match. In addition, we used a nationally representative family survey that included detailed information on personal health care utilization and out-of-pocket medical services, methods of financing out-of-pocket medical expenditures, and information on individual level labor force participation. Finally, our results rely on multiple robustness tests. Our results apply to different matching methods, as well as to families that have died of any cause, and to the top 1% of families that spend the most out of pocket on ECD treatment.

## 5. Conclusions

This study measured the economic burden of elderly chronic disease (ECD) in Chinese families using the China Health and Retirement Longitudinal Study (CHARLS) data set from Peking University (China). We studied some aspects of this burden, including health-service utilization, family expenditures, and labor force participation rates of family members. Our results suggest that the economic ECD burden-whether it is public subsidies or out-of-pocket spending by families-may not be as great as people conclude in the absence of a match. Inevitably, our study also has some limitations, which will be studied in the future. (a) The matching methods do not explain unobservable factors that contribute to a family’s ECD risk. For example, information on alcohol and tobacco consumption, dietary history or family obesity, and family members’ professional histories. The estimated economic ECD burden could move upward if this information is not available. (b) Biased estimates can also occur because we do not have information about differences in physical contacts, such as distance to health facilities, that could affect ECD diagnosis and any associated health expenditures. (c) The survey also did not collect information about the severity of ECD. If the information on severity is available, we can use a variant of the propensity score matching approach to evaluate multiple treatment cases, assessing the financial burden of families based on severity. In the absence of this information, we can only estimate the average burden of different ECD severity. (d) From an economic perspective, we have not taken into account the temporal evolution of inflation and economic growth.

## Figures and Tables

**Table 1:** Summary of outcome variables by ECD-family and control family.

Outcome Variable	ECD-Family	Matched-Control Family	Unmatched-Control Family
Health service utilization (per family member)			
Hospitalization rate (1 year)	0.234 (0.25, 0.28)	0.109 * (0.11, 0.13)	0.087 (0.095, 0.098)
Public Hospitalization rate (1 year)	0.129 (0.13, 0.5)	0.051 * (0.05, 0.07)	0.047 (0.0438, 0.0453)
Length of hospital stay (in days) (1 year)	4.556 (3.78, 4.69)	1.131 * (0.92, 1.43)	0.934 (0.909, 0.922)
Outpatient visits (15 days)	0.199 (0.17, 0.21)	0.143 * (0.14, 0.17)	0.122 (0.119, 0.122)
Outpatient visits of non-ECD patients (15 days)	0.109 (0.11, 0.14)	0.148 * (0.14, 0.17)	0.118 (0.118, 0.121)
Outpatient visits for non-major conditions (15 days)	0.081 (0.05, 0.07)	0.117 * (0.11, 0.15)	0.097 (0.101, 0.098)
Consumption (per family member)			
Inpatient OP expenditure (CNY) (1 year)	7556 (5564, 9111)	2,148 * (1431, 2908)	1528 (1466, 1576)
Outpatient OP expenditure (CNY) (15 days)	117.31 (91.12, 144.55)	48.17 * (34.95, 57.39)	35.17 (34.02, 35.35)
Outpatient OP expenditure (CNY) on non-major health conditions (15 days)	16.58 (11.17, 19.93)	38.48 * (24.17, 42.4)	24.25 (23.45, 25.23)
Outpatient OP expenditure (CNY) for members without ECD (15 days)	29.12 (27.21, 36.88)	46.17 * (34.95, 57.39)	34.15 (33.02, 35.25)
Non-medical consumption expenditure (CNY) (15 days)	324 (289, 412)	422 (402, 442)	422 (419, 424)
PCT of family reporting borrowing or selling assets to finance inpatient care (1 year)	52.41 (48.98, 55.82)	16.77 * (14.28, 19.26)	16.71 (16.46, 16.92)
PCT of family reporting borrowing/selling assets to finance outpatient care (15 days)	7.47 (5.79, 9.15)	4.11 * (2.12, 4.69)	3.45 (3.34, 3.55)
Workforce Participation			
PCT of family members aged 45+ who are working	49.51 (47.68, 51.32)	51.91 (48.88, 53.66)	54.79 (54.57, 54.98)
PCT of family members without ECD aged 45+ who are working	53.58 (51.30, 55.86)	51.91 (48.11, 52.57)	54.84 (54.64, 55.11)

Note: PCT = Percentage. CNY = China Yuan, OP = Out-of-pocket. The data presented refer to all family, regardless of whether there was a death in the household. 95% confidence intervals are reported in parentheses underneath the means for each statistic. * indicates that the treatment and matched controls are significantly different at the 5% level.

**Table 2:** Effects of elderly chronic disease (ECD) on family health service utilization in China. (Unit: per family member).

Outcome Index	Matched-Family (by Nearest Neighbor)	Matched-Family (by Stratification)	Excluding Family with Death (by Nearest Neighbor)	Excluding Family with 1% Most Expensive ECD Cases (by Nearest Neighbor)
Hospitalization rate (1 year)	0.264 (<0.01)	0.276 (<0.01)	0.265 (<0.01)	0.257 (<0.01)
Public Hospitalization rate (1 year)	0.184 (<0.01)	0.185 (<0.01)	0.175 (<0.01)	0.182 (<0.01)
Length of hospital stay (1 year)	3.316 (<0.01)	3.479 (<0.01)	3.424 (<0.01)	3.403 (<0.01)
Outpatient visits (15 days)	0.067 (<0.01)	0.079 (<0.01)	0.099 (<0.01)	0.068 (<0.01)
Public outpatient visits (15 days)	0.037 (<0.01)	0.045 (<0.01)	0.042 (<0.01)	0.033 (<0.01)
Outpatient visits of non-ECD patients (15 days)	−0.045 (<0.01)	−0.034 (0.013)	−0.028 (0.146)	−0.043 (0.01)
Outpatient visits for non-major health conditions, (15 days)	−0.056 (<0.01)	−0.042 (<0.01)	−0.038 (<0.01)	−0.045 (<0.01)

Notes: Values in parentheses refer to *p*-values that the matched ECD-affected and control outcomes differ in a two-tailed test. ‘Non-major’ health conditions refer to all health conditions except cancer, heart disease, stroke, injuries and diabetes.

**Table 3:** Robustness check-effects of ECD on health service utilization in China. (Unit: per family member).

Outcome Index	Matched-Family (by Nearest Neighbor)	Matched-Family (by Stratification)	Excluding Family with Death (by Nearest Neighbor)	Excluding Family with 1% Most Expensive ECD Cases (by Nearest Neighbor)
Hospitalization rate (1 year)	0.048 (<0.01)	0.047 (<0.01)	0.033 (0.013)	0.045 (<0.01)
Public Hospitalization rate (1 year)	0.023 (0.03)	0.029 (<0.01)	0.020 (0.058)	0.025 (0.017)
Length of hospital stay (1 year)	2.079 (<0.01)	2.164 (<0.01)	2.048 (<0.01)	2.035 (<0.01)
Outpatient visits (15 days)	0.066 (<0.01)	0.064 (<0.01)	0.066 (<0.01)	0.047 (<0.01)
Public outpatient visits (15 days)	0.037 (<0.01)	0.037 (<0.01)	0.044 (<0.01)	0.038 (<0.01)
Outpatient visits of non-ECD patients (15 days)	−0.035 (<0.01)	−0.039 (<0.01)	−0.039 (<0.01)	−0.053 (<0.01)
Outpatient visits for non-major health conditions, (15 days)	−0.042 (<0.01)	−0.042 (<0.01)	−0.048 (<0.01)	−0.055 (<0.01)

Notes: Robustness check refers to propensity scores generated with a hospitalization. Values in parentheses refer to *p*-values that the matched ECD-affected and control outcomes differ in a two-tailed test. ‘Non-major’ health conditions refer to conditions excluding cancer, heart disease, stroke, injuries and diabetes.

**Table 4:** Effects of ECD on family consumption and workforce participation in China. (Unit: CNY per family member).

Outcome Index	Matched-Family (by Nearest Neighbor)	Matched-Family (by Stratification)	Excluding Family with Death (by Nearest Neighbor)	Excluding Family with 1% most Expensive ECD Cases (by Nearest Neighbor)
Household Consumption				
Inpatient OP expenditure (1 year)	5233.07 (<0.01)	5438.66 (<0.01)	5045.31 (<0.01)	4576.75 (<0.01)
Outpatient OP expenditure (15 days)	74.18 (<0.01)	79.96 (<0.01)	86.34 (<0.01)	66.68 (<0.01)
Outpatient OP expenditure on non-major health conditions (15 days)	−18.95 (<0.01)	−13.83 (<0.01)	−11.99 (<0.01)	−12.96 (<0.01)
Outpatient OP expenditure for members without ECD (15 days)	−18.13 (0.01)	−12.42 (<0.01)	−11.56 (0.05)	−14.79 (0.03)
Non-medical consumption expenditure (15 days)	−27.69 (0.265)	−48.79 (0.020)	−85.63 (0.033)	−33.15 (0.174)
PCT of borrowing or selling assets to finance inpatient care (1 year)	0.357 (<0.01)	0.362 (<0.01)	0.393 (<0.01)	0.336 (<0.01)
PCT of borrowing/selling assets to finance outpatient care (15 days)	0.034 (<0.01)	0.042 (<0.01)	0.038 (<0.01)	0.032 (<0.01)
Workforce Participation				
PCT of family members aged 45+ who are working	−2.41 (0.07)	−3.03 (<0.01)	−2.81 (<0.07)	−3.32 (0.03)
PCT of family members without ECD aged 45+ who are working	1.71 (0.26)	1.11 (<0.36)	1.92 (0.26)	0.72 (0.59)

Notes: CNY = China Yuan, OP = Out-of-pocket. Values in parentheses refer to *p*-values that the matched ECD-affected and control outcomes differ in a two-tailed test. ‘Non-major’ health conditions refer to conditions other than cancer, heart disease, stroke, injuries and diabetes.

**Table 5:** Robustness check-effect of ECD on family consumption and workforce participation in China. (Unit: CNY per family member).

Outcome Index	Matched-Family (by Nearest Neighbor)	Matched-Family (by Stratification)	Excluding Family with Death (by Nearest Neighbor)	Excluding Family with 1% Most Expensive ECD Cases (by Nearest Neighbor)
Household Consumption				
Inpatient OP expenditure (1 year)	4403.95 (<0.01)	4401.53 (<0.01)	3942.24 (<0.01)	3534.73 (<0.01)
Outpatient OP expenditure (15 days)	82.15 (<0.01)	76.32 (<0.01)	85.43 (<0.01)	59.51 (<0.01)
Outpatient OP expenditure on non-major health conditions (15 days)	−17.53 (<0.04)	−21.82 (<0.01)	−19.02 (<0.01)	−29.98 (<0.01)
Outpatient OP expenditure for members without ECD (15 days)	−19.16 (<0.05)	−24.95 (<0.01)	−29.89 (<0.01)	−19.94 (<0.01)
Non-medical consumption expenditure (15 days)	−17.91 (0.507)	−37.27 (0.095)	−45.23 (0.089)	−38.16 (0.166)
PCT of borrowing or selling assets to finance inpatient care (1 year)	0.172 (<0.01)	0.168 (<0.01)	0.156 (<0.01)	0.138 (<0.01)
PCT of borrowing/selling assets to finance outpatient care (15 days)	0.029 (0.01)	0.033 (<0.01)	0.036 (<0.01)	0.021 (<0.073)
Workforce Participation				
PCT of family members aged 45+ who are working	−0.51 (0.683)	−1.78 (0.051)	−2.42 (0.088)	−2.03 (0.127)
PCT of family members without ECD aged 45+ who are working	3.61 (0.018)	2.32 (0.052)	2.22(0.174)	2.05 (0.168)

Notes: CNY = China Yuan, OP = Out-of-pocket. Robustness check refers to propensity-score matching that included hospitalization. CNY = Indian Rupees. Values in parentheses refer to *p*-values that the matched ECD-affected and control outcomes differ in a two-tailed test. ‘Non-major’ health conditions refer to conditions other than cancer, heart disease, stroke, injuries and diabetes.

## Abbreviations

ECD: Chronic Diseases among the elderly, CHARLS: China Health and Retirement Longitudinal Study, GDP: Gross domestic product, PSM: Propensity score matching, CHSY: China health statistics yearbook, PCT: Percentage, OP: Out-of-pocket.

## References

[1] WHO (2012). GLOBOCAN. Estimated Cancer Incidence, Mortality and Prevalence Worldwide in 2012. http://globocan.iarc.fr.

[2] Ferlay J., Shin H.R., Bray F., Forman D., Mathers C., Parkin D.M. (2010). Estimates of worldwide burden of cancer in 2008: GLOBOCAN 2008. Int. J. Cancer.

[3] Murray C.J., Ezzati M., Flaxman A.D., Lim S., Lozano R., Michaud C., Naghavi M., Salomon J.A., Shibuya K., Vos T. (2012). GBD 2010: Design, definitions, and metrics. Lancet.

[4] Pi T., Wu H., Li X. (2019). Does Air Pollution Affect Health and Medical Insurance Cost in the Elderly: An Empirical Evidence from China. Sustainability.

[5] Popkin B., Horton S., Kim S., Mahal A., Shuigao J. (2001). Trends in diet, nutritional status, and diet-related non-communicable diseases in China and India: The economic costs of the nutrition transition. Nutr. Rev..

[6] National Bureau of Statistics of China (2017). China Population and Employment Statistics Yearbook.

[7] Xu X., Chen L. (2019). Influencing factors of disability among the elderly in China, 2003–2016: Application of Bayesian quantile regression. J. Med. Econ..

[8] Bloom D., Cafiero E., Jane-Llopis E., Abrahams-Gessel S., Bloom L., Fathima S., Feigl A.B., Gaziano T., Mowafi M., Pandya A. (2011). The Global Economic Burden of Non-Communicable Diseases.

[9] Abegunde D.O., Mathers C.D., Adam T., Ortegon M., Strong K. (2007). The burden and costs of chronic diseases in low-income and middle-income countries. Lancet.

[10] Wong G., Lim W.H., Lewis J., Craig J.C., Turner R., Zhu K., Lim E.M., Prince R. (2015). Vitamin D and cancer mortality in elderly women. BMC Cancer.

[11] United Nations (2017). DESA/Population Division “World Population Prospects: The 2017 Revision, Key Findings and Advance Tables”.

[12] Xu X., Chen L. (2019). Projection of Long-Term Care Costs in China, 2020–2050, Based on the Bayesian Quantile Regression Method. Sustainability.

[13] Shugang L., Xuefei Z., Yizhong Y., Kui W., Dongsheng R., Lijuan P., Feng L. (2015). High Cancer Burden in Elderly Chinese, 2005–2011. Int. J. Environ. Res. Public Health.

[14] Xu X., Xu Z., Chen L., Li C. (2019). How Does Industrial Waste Gas Emission Affect Health Care Expenditure in Different Regions of China: An Application of Bayesian Quantile Regression. Int. J. Environ. Res. Public Health.

[15] Liu J., Rozelle S., Xu Q., Yu N., Zhou T. (2019). Social Engagement and Elderly Health in China: Evidence from the China Health and Retirement Longitudinal Survey (CHARLS). Int. J. Environ. Res. Public Health.

[16] Johnson C.M., Wei C., Ensor J.E., Smolenski D.J., Amos C.I., Levin B., Berry D.A. (2013). Meta-analyses of colorectal cancer risk factors. Cancer Cause Control.

[17] Long M. (2013). The restriction of population aging on the population development strategy of China and the solution. Popul. Dev..

[18] Chen W., Zheng R., Zeng H., Zhang S. (2014). Trend analysis of the changes of male/female, urban/rural incidences and average age of cancer patients in China 1989–2008. Chin. J. Oncol..

[19] Chen W., Zheng R., Zeng H., Zhang S., He J. (2015). Annual report on status of cancer in China, 2011. Chin. J. Cancer Res..

[20] Jiang J., Huang W., Wang Z., Zhang G. (2019). The Effect of Health on Labour Supply of Rural Elderly People in China—An Empirical Analysis Using CHARLS Data. Int. J. Environ. Res. Public Health.

[21] Australian Institute of Health and Welfare (2012). Australian Health Statistics 2010–2011.

[22] Gertler P., Gruber J. (2002). Insuring consumption against illness. Am. Econ. Rev..

[23] Islam A., Maitra P. (2012). Health shocks and consumption smoothing in rural households: Does microcredit have a role to play?. J. Dev. Econ..

[24] Wagstaff A. (2008). Measuring Financial Protection in Health.

[25] Lilly M., Laporte A., Coyte P. (2010). Do they care too much to work? The influence of caregiving intensity on the labour force participation of unpaid caregivers in Canada. J. Health Econ..

[26] Ciani E. (2011). Informal Adult Care and Caregivers’ Employment in Europe. Labour Econ..

[27] Shaw W., Patterson T., Semple S., Ho S., Irwin M.R., Hauger R.L., Grant I. (1997). Longitudinal analysis of multiple indicators of health decline among spousal caregivers. Ann. Behav. Med..

[28] Roberts A. (1992). The labor market consequences of family illness. J. Ment. Health Policy Econ..

[29] Rosenbaum P., Rubin D. (1983). The central role of the propensity score in observational studies for causal effects. Biometrika.

[30] Ajay M., Anup K., Victoria Y.F., Michael E. (2013). The Economic Burden of Cancers on Indian Households. PLoS ONE.

[31] Wen X.X., Wen F., Ye L.X. (2017). The effects of social capital on mental health of the Chinese rural elderly: An analysis based on survey data from the China Health and Retirement Longitudinal Study. China Rural. Surv..

[32] Yamano T., Jayne T. (2004). Measuring the impacts of working-age adult mortality on small-scale farm households in Kenya. World Dev..

